# Integrative analysis of AT classification, plasma biomarkers, and cognitive trajectories across diverse dementia syndromes

**DOI:** 10.1038/s41598-025-30117-y

**Published:** 2025-12-11

**Authors:** Jihwan Yun, Min Young Chun, Henrik Zetterberg, Kaj Blennow, Fernando Gonzalez-Ortiz, Nicholas J. Ashton, Daeun Shin, Soyeon Yoon, Heejin Yoo, Jun Pyo Kim, Hongki Ham, Yuna Gu, Hee Jin Kim, Seung Hwan Moon, Hanna Cho, Jae Yong Choi, Byung Hyun Byun, Su Yeon Park, Jeong Ho Ha, Duk L. Na, Sang Won Seo, Hyemin Jang

**Affiliations:** 1 Department of Neurology, Kyung Hee University Hospital, Kyung Hee University College of Medicine, Seoul, South Korea; 2 Department of Neurology, Yonsei University College of Medicine, Seoul, South Korea; 3 Department of Neurology, Yongin Severance Hospital, Yonsei University Health System, Yongin, South Korea; 4 Department of Psychiatry and Neurochemistry, Institute of Neuroscience and Physiology, the Sahlgrenska Academy at the University of Gothenburg, Gothenburg, Sweden; 5 Clinical Neurochemistry Laboratory, Sahlgrenska University Hospital, Gothenburg, Sweden; 6 Department of Neurodegenerative Disease, UCL Institute of Neurology, Queen Square, London, UK; 7 UK Dementia Research Institute at UCL, London, UK; 8 Hong Kong Center for Neurodegenerative Diseases, InnoHK, Clear Water Bay, Hong Kong, China; 9 Wisconsin Alzheimer’s Disease Research Center, School of Medicine and Public Health, University of Wisconsin, University of Wisconsin-Madison, Madison, WI 53792 USA; 10 Pitié-Salpêtrière Hospital, Paris Brain Institute, ICM, Sorbonne University, Paris, France; 11 Neurodegenerative Disorder Research Center, Division of Life Sciences and Medicine, Department of Neurology, Institute on Aging and Brain Disorders, University of Science and Technology of China and First Affiliated Hospital of USTC, Hefei, P.R. China; 12 King’s College London, Institute of Psychiatry, Psychology and Neuroscience, Maurice Wohl Clinical Neuroscience Institute, London, UK; 13 NIHR Biomedical Research Centre for Mental Health and Biomedical Research Unit for Dementia at South London and Maudsley NHS Foundation, London, UK; 14 Centre for Age-Related Medicine, Stavanger University Hospital, Stavanger, Norway; 15 Alzheimer’s Disease Convergence Research Center, Samsung Medical Center, Seoul, South Korea; 16 Department of Neurology, Samsung Medical Center, Sungkyunkwan University School of Medicine, 81 Irwon-ro, Gangnam-gu, Seoul, 06351 Republic of Korea; 17 Department of Health Sciences and Technology, SAIHST, Sungkyunkwan University, Seoul, South Korea; 18 Department of Nuclear Medicine, Samsung Medical Center, Sungkyunkwan University School of Medicine, Seoul, South Korea; 19 Department of Neurology, Gangnam Severance Hospital, Yonsei University College of Medicine, Seoul, 135-720 Korea; 20 Division of Applied RI, Korea Institute of Radiological & Medical Sciences, Seoul, Korea; 21 Department of Nuclear Medicine, Institute of Radiological & Medical Sciences, Korea Cancer Center Hospital, Seoul, Korea; 22 Department of Neurology, Korea Cancer Center Hospital, Korea Institute of Radiological & Medical Sciences, Seoul, Korea; 23 Happymind Clinic, Seoul, South Korea; 24 Department of Neurology, Asan Medical Center, University of Ulsan College of Medicine, 88 Olympic-ro 43-gil, Songpa-gu, Seoul, 05505 Republic of Korea

**Keywords:** Amyloid-β, Tau, Alzheimer’s disease, Subcortical vascular cognitive impairment, Frontotemporal dementia, Positron emission tomography, Biomarkers, Diseases, Neurology, Neuroscience

## Abstract

This study aimed to examine amyloid-β (Aβ) and tau (AT) biological stages, plasma biomarkers, and cognitive trajectories according to AT stages in Alzheimer’s disease-related cognitive impairment (ADCI), subcortical vascular cognitive impairment (SVCI), and frontotemporal dementia (FTD). A total of 275 participants (42 cognitively unimpaired [CU], 132 ADCI, 73 SVCI, and 28 FTD) underwent Aβ and tau positron emission tomography for assessment of AT stages. Participants with cognitive impairment (ADCI, SVCI, and FTD) were classified according to clinical stages of mild cognitive impairment or dementia. Plasma biomarkers were analysed, and cognitive trajectories were assessed using mixed-effects models over 6.1 years. SVCI and FTD showed more advanced clinical stages than ADCI at equivalent AT stages. SVCI participants had higher plasma glial fibrillary acidic protein (*P* = 0.012) and neurofilament light chain (*P* = 0.025) than CU participants in the A − T− stage. In the A − T− stage, SVCI (β = -0.738, *P* = 0.002) and FTD (β = -4.016, *P* < 0.001) showed faster cognitive decline than CU, but not ADCI. In the A + T − stage, ADCI (β = -0.634, *P* = 0.005) and SVCI (β = -0.690, *P* = 0.006) showed faster decline than CU. In the A + T + stage, only ADCI exhibited significantly faster decline than CU (β = -1.856, *P* = 0.008). Distinct plasma biomarker profiles and cognitive trajectories characterise ADCI, SVCI, and FTD across AT classification, highlighting the heterogeneity in pathophysiological mechanisms across dementia types.

**Supplementary Information:**

The online version contains supplementary material available at 10.1038/s41598-025-30117-y.

## Introduction

Alzheimer’s disease (AD) research has accelerated the development of biomarkers, enabling the establishment of biological staging that reflects the progression of β-amyloid (Aβ) and tau pathologies in the brain^1^. Biological staging can help categorise disease progression even before clinical symptoms emerge. Efforts are currently underway to integrate biological staging with clinical stages—cognitively unimpaired (CU), mild cognitive impairment (MCI), and dementia—to more comprehensively link AD pathology to clinical progression in Alzheimer’s clinical syndrome^2^.

Previous studies have reported the presence of AD biomarkers in non-AD dementias as well^3–5^. Specifically, most studies used Aβ (^11^C- and ^18^F-labeled ligands) and tau (^18^F-flortaucipir) positron emission tomography (PET) imaging, which allowed for assessing topographical patterns and quantifying AD pathologies. Tau PET has been acknowledged as the modality capable of detecting and assessing the topographical pattern of AD-specific tau pathology in vivo, although plasma biomarkers, such as MTBR-tau243, have been developed to detect AD tau proteinopathy^2^. Among non-AD dementias, subcortical vascular cognitive impairment (SVCI), characterized by severe cerebral small vessel disease, has been of significant interest due to its frequent coexistence and interaction with AD pathology. Studies have shown that 30 ~ 70% of SVCI participants (*n* ranging from 45 to 583) exhibit Aβ uptake on PET^3,6,7^, which contributes to faster clinical progression^8–10^. We also applied Aβ/tau classification scheme to SVCI (*n* = 60)^10,11^, demonstrating its potential value as a prognostic marker. Frontotemporal dementia (FTD) represents another distinct clinical entity among non-AD dementias. While AD biomarker studies in FTD are less prevalent than those in SVCI, several studies indicate that a subset (10 ~ 20%) of FTD participants (*n* ranging from 94 from 288) demonstrate Aβ biomarkers^3,12,13^. Although some clinical cases of FTD may have underlying AD pathologies, Aβ and tau PET have been primarily used to differentiate FTD from AD^14–16^. Importantly, however, previous studies have not integrated AD biological and clinical staging in non-AD dementias. Thus, the distribution of AD biological stages and its significance regarding cognitive decline across different dementia subtypes remain less well understood.

Recently, plasma AD biomarkers, such as phosphorylated-tau (p-tau), glial fibrillary acidic protein (GFAP), and neurofilament light chain (NfL), have been developed to reflect core AD pathology, neuroinflammation, and neurodegeneration, respectively^17^. Notably, GFAP and NfL are also associated with non-AD processes due to their nonspecific characteristics. Investigating the distinct elevations of various plasma biomarkers according to AD biological stages in non-AD dementia may provide insights into potential interactions or synergies between AD biomarkers and other pathologies. Therefore, understanding how AD biological staging interacts with other pathological processes in non-AD dementia could provide valuable insights into disease mechanisms and progression.

In this study, we aimed to examine the distribution of AD biological stages—AT stages defined by Aβ and tau PET positivity—and their characteristics and impact on cognitive decline across different dementia subtypes. First, we examined how the distribution of AT stages varies across clinical stages in AD-related cognitive impairment (ADCI), SVCI, and FTD. Second, we assessed differences in plasma biomarker levels according to diagnosis and AT stages. Finally, we explored how cognitive trajectories differ by AT stage in these groups to gain insights into the relationship between AD biological stages and clinical progression across different types of dementia.

## Methods

### Participants

A total of 275 participants, including 42 CU, 132 ADCI, and non-ADCI (73 SVCI and 28 FTD) participants, aged 55–90 years, were recruited as part of a sub-study of the Korea-Registries to Overcome Dementia and Accelerate Dementia Research (K-ROAD) project in South Korea^18^. The K-ROAD project is a multicenter initiative aiming to facilitate the development of advanced diagnostic and therapeutic techniques for neurodegenerative dementias. For this study, we exclusively recruited individuals from the memory clinic of Samsung Medical Center where all participants underwent a single set of standardized protocols for all assessments, including imaging scans, ensuring consistency across the study population.

All participants underwent neuropsychological testing, *APOE* genotyping, brain magnetic resonance imaging (MRI), Aβ PET using ^18^F-florbetaben or ^18^F-flutemetamol, and tau PET using ^18^F-flortaucipir. CU individuals met the following criteria^19^: (1) no medical history likely to affect cognitive function, based on Christensen’s health screening criteria; (2) no objective cognitive impairment, as determined by a comprehensive neuropsychological test battery, with scores above − 1.0 standard deviations (SD) of age- and education-matched norms in memory and above − 1.5 SD in other cognitive domains^20^. The ADCI group included cognitively impaired individuals with Alzheimer’s clinical syndrome, regardless of Aβ biomarker status, encompassing MCI and dementia of Alzheimer’s type. In contrast, the SVCI group comprised participants with severe ischemia, including those clinically diagnosed with subcortical vascular mild cognitive impairment and subcortical vascular dementia. Severe ischemia was identified on brain MRI, defined by periventricular white matter hyperintensities (WMH) > 10 mm and deep WMH > 25 mm, as modified from the Fazekas ischaemia criteria^21,22^. Participants meeting this criterion for severe WMH were explicitly excluded from the ADCI and FTD cohorts. Moreover, participants with FTD syndromes included those with a clinical diagnosis of behavioural variant FTD^23^, semantic variant primary progressive aphasia, nonfluent/agrammatic variant primary progressive aphasia^24^, corticobasal syndrome^25^, progressive supranuclear palsy syndrome^26^, and FTD–motor neuron disease^27^, diagnosed based on the respective diagnostic criteria for each condition. All FTD syndromes were diagnosed based on the patient’s clinical course, neurologic examination, neuropsychological testing, and brain imaging. The FTD cohort was composed of participants with a range of clinically diagnosed frontotemporal syndromes. and encompassed individuals at both the MCI and dementia stages to capture the full continuum of these progressive disorders.

All participants in the ADCI, SVCI, and FTD groups were classified into MCI and dementia. MCI was defined by the following criteria^28^: (1) subjective cognitive complaints from the patient or caregiver; (2) objective cognitive impairment; (3) no significant impairment in activities of daily living (ADL); and (4) no dementia. Dementia was defined as the presence of objective cognitive impairment accompanied by impairment in ADL. The criterion for objective cognitive impairment varied between groups. In the ADCI group, it was defined as performance below the 16th percentile in memory domains^29^. In the SVCI group, it was defined as performance below the 16th percentile in any domain—including language, visuospatial, memory, or frontal function—on neuropsychological testing. In the FTD group, characterised by a clinical diagnosis of FTD syndromes, participants were classified as having MCI if ADL were preserved and the Clinical Dementia Rating (CDR) was 0.5, or as having dementia if ADL were impaired and the CDR was 1 or higher.

The institutional review board of Samsung Medical Center (No. 2020–12–177) approved this study. Informed consent was obtained from all subjects and/or their legal guardian(s). All methods were performed in accordance with the relevant guidelines and regulations, including the Declaration of Helsinki.

### Aβ PET acquisition and definition of Aβ positivity

For ^18^F-florbetaben and ^18^F-flutemetamol PET, a 20-min dynamic emission PET scan (divided into four 5-min segments) was obtained 90 min after administration of a mean dose of 311.5 MBq of florbetaben or 197.7 MBq of flutemetamol. The resulting three-dimensional PET images were reconstructed using the ordered-subset expectation maximisation algorithm, producing a 128 × 128 × 48 matrix with voxel dimensions of 2 × 2 × 3.27 mm (florbetaben: 4 iterations, 20 subsets; flutemetamol: 4 iterations, 20 subsets).

Aβ uptake was measured using a regional direct comparison Centiloid (rdcCL) method developed in our previous study, which harmonizes florbetaben and flutemetamol PET ligands without requiring ^11^C-labelled Pittsburgh compound B images^30^. PET images were registered to the MRI for anatomical alignment. The target volume of interest (VOI) was defined as the standard centiloid global cortical target VOI from the Global Alzheimer’s Association Interactive Network website (http://www.gaain.org), and the whole cerebellum was set as the reference region. Aβ PET positivity (A+) was defined using a global MRI-based rdcCL threshold of 25.5^31^, which was obtained using the Gaussian mixture model from 3,753 participants aged 55 years and older who underwent florbetaben or flutemetamol PET^31,32^. The global MRI-based rdcCL scales for both PET imagings showed an area under the curve > 0.9 for Aβ PET positivity by visual assessment. All imaging analyses were conducted at the laboratory of Samsung Medical Center.

### Tau PET acquisition and definition of tau positivity

A series of scans were conducted 80 min after administering a typical 280 MBq dose of ^18^F-flortaucipir. The procedure involved a 20-min dynamic emission PET scan, consisting of four 5-min frames. The three-dimensional PET images were reconstructed using the ordered-subset expectation maximisation algorithm into a 256 × 256 × 223 matrix (6 iterations, 16 subsets), with a voxel size of 1.591 × 1.591 × 1 mm. These images were aligned with individual MRIs and standardised to a T1-weighted MRI template. The transformed PET images were normalised using cerebellar grey matter as a baseline. Standardised uptake value ratio (SUVR) values were then spatially smoothed using an 8-mm Gaussian kernel, and the data were processed using the Mayo Clinic Adult Lifespan Template atlas. For tau uptake analysis, temporal meta-regions of interest (ROIs) were defined, including the entorhinal cortex, amygdala, fusiform gyrus, parahippocampal gyrus, and the inferior and middle temporal gyri. A tau PET positivity (T+) threshold was set at an SUVR of 1.38 in these ROIs, which is 2 SD above the mean SUVR of the same regions in Aβ PET-negative CU participants (*n* = 17)^18^. Additionally, tau uptakes were also calculated from two sets of ROIs: (1) medial temporal lobe regions, including the entorhinal cortex and amygdala; and (2) neo-temporal regions, including the inferior and middle temporal gyri.

### Plasma collection and processing

A total of 173 participants (29 CU; 99 ADCI; 39 SVCI; and 6 FTD) underwent plasma biomarker analysis. Blood (8 mL) was drawn from each participant and placed in tubes containing 0.5 Methylenediaminetetraacetic acid. The samples were agitated for 5 min and then centrifuged at 1300×g for 10 min, separating the plasma into 5–10 vials, each containing 0.3 mL. The plasma samples were stored at −75 °C until analysis. All procedures were conducted in accordance with the guidelines set by the National Biobank of South Korea for human resource collection and registration. The interval between plasma collection and Aβ PET scan ranged from 0 to 69 days, with a mean of 4 days.

Plasma samples were transported in a frozen state at −70 °C to the Department of Psychiatry and Neurochemistry, University of Gothenburg, for analysis. Upon arrival, the samples were thawed on wet ice and centrifuged at 500×g for 5 min at 4 °C. Plasma GFAP and NfL concentrations were measured using the commercial Neurology 4-Plex E kit (Quanterix, Billerica, MA, USA). For plasma p-tau, specifically p-tau at threonine 217 (p-tau217), we used the commercial ALZPath p-tau217 assay kit. The measurements were performed by analysts blinded to the clinical data, using one round of experiments with a single batch of reagents. The intra-assay coefficients of variation for the biomarker assays were < 10%. Plasma biomarker analyses included CU, ADCI, and SVCI; the FTD subgroup (*n* = 6) was excluded due to insufficient sample size for reliable estimates.

### Brain MRI acquisition

All participants underwent brain MRI using a standardised imaging protocol, including three-dimensional T1 turbo field echo imaging and fluid-attenuated inversion recovery (FLAIR), on a 3.0 Tesla MRI scanner. T1-weighted images were acquired on all scanners with a standard isotropic voxel size of 1 mm.

### AT biological and clinical staging

Participants were categorised into A − T−, A + T−, and A + T + stages based on Aβ and tau PET positivity, and clinically staged as CU, MCI, or dementia.

### Longitudinal follow-up

The longitudinal study included 262 participants (41 CU, 125 ADCI, 70 SVCI, and 26 FTD) who underwent two or more assessments using the Korean version of the Mini-Mental State Examination (MMSE)^33^. We obtained both retrospective and prospective relative to the time of tau PET scans, with a mean follow-up period of 6.09 years (range, 0.51 to 20.90 years). The groups with very small samples in FTD (A + T−, *n* = 1; A + T+, *n* = 2) were excluded to avoid unstable estimates.

### Statistical analysis

The frequencies and proportions of participants classified as A + or T + were calculated for each diagnostic group. The distribution of AT biological and clinical stages in ADCI, SVCI, and FTD, compared with that in CU, was presented using heatmaps. Plasma biomarker analyses were performed after log-transformation due to the non-normality of the data.

Differences in PET uptake were assessed with analysis of covariance (ANCOVA), adjusting for only age in AT-stage comparisons, followed by Tukey-corrected post hoc pairwise comparisons. Differences in plasma biomarker levels were also evaluated using ANCOVA. For comparisons between diagnostic groups within each AT stage, models included age, *APOE* genotype, and tau SUVR in the neo-temporal ROI as covariates. For comparisons between AT stages within each diagnostic group, age and *APOE* genotype were included as covariates. Tukey-corrected post hoc pairwise comparisons were applied in all models.

To investigate the effects of co-pathologies on cognitive trajectories in non-ADCI groups (when AT biological stages were identical across groups), we conducted linear mixed-effects models with random intercepts and random slopes over time within each AT stage. Models were adjusted for age, education level, *APOE* genotype, and tau SUVR in neo-temporal ROI as covariates (fixed effects: diagnostic group, time, diagnostic group*time, covariates). In this model, ‘time’ was defined as the number of years from the date of the tau PET scan (Time 0), and all available MMSE scores (retrospective and prospective) were included; the mean follow-up was 6.09 years with an individual range of 0.51 to 20.90 years. Additionally, when comparing cognitive trajectories across AT biological stages within the same clinical diagnosis, similar linear mixed-effects models were applied, adjusting for age, education level, and *APOE* genotype as covariates (without including tau SUVRs). A false discovery rate (FDR) of 0.05 was used to correct for multiple comparisons. Continuous variables are expressed as the median (interquartile range, 25th − 75th percentile), while categorical variables are expressed as frequency (percentage). For cognitive trajectories by AT stages within each diagnostic group, we fit separate linear mixed-effects models within each diagnostic group (CU, ADCI, SVCI) with MMSE as the outcome and an interaction between time and AT stage (A − T−, A + T−, A + T+), adjusted for age, education level, and *APOE* genotype. All analyses were performed using SAS version 9.4 (SAS Institute Inc., Cary, NC, USA), R version 4.3.2, and R Studio version 2023.12.0 + 369. Statistical significance was set at *P* < 0.05 for two-tailed tests.

## Results

### Characteristics of study participants

Table 1 presents the demographic characteristics: the median age was 78 years for SVCI (*n* = 73), 63 years for FTD (*n* = 28), and 73 years for ADCI (*n* = 132). The proportion of participants in the dementia stage was 56.2% (47/73) in SVCI, 64.3% (18/28) in FTD, and 61.4% (81/132) in ADCI. Aβ PET positivity was observed in 49.3% (36/73) of SVCI, 10.7% (3/28) of FTD, and 93.9% (124/132) of ADCI. For CU (*n* = 42), the median age was 75 years, with 50.0% (21/42) showing Aβ PET positivity.

**Table 1:** Demographic and clinical characteristics of the study population.

	CU (*N* = 42)	ADCI (*N* = 132)	SVCI (*N* = 73)	FTD (*N* = 28)	*P* value
Age, y	75.0 [67.0;76.0]	73.0 [66.0;78.0]	78.0 [74.0;83.0]	63.0 [59.5;72.0]	< 0.001
Female, *n* (%)	24 (57.14%)	49 (37.1%)	21 (28.7%)	14 (50.0%)	0.014
Education, y	12.0 [6.0;16.0]	12.00 [9.0;16.0]	6.0 [3.0;12.0]	12.0 [9.0;16.0]	< 0.001
*APOE* ε4 carriers (%)	12 (28.6%)	73 (55.3%)	26 (35.6%)	9 (33.3%)	0.003
MMSE	28.5 [28.0;30.0]	20.0 [14.5;24.0]	21.0 [18.0;26.0]	19.5 [6.0;25.5]	< 0.001
Aβ PET positivity	21 (50.0%)	124 (93.9%)	36 (49.3%)	3 (10.7%)	< 0.001
Tau PET positivity	5 (11.9%)	91 (68.9%)	14 (19.2%)	2 (7.1%)	< 0.001
Dementia, n (%)	NA	81 (61.4%)	41 (56.2%)	18 (64.3%)	< 0.001
P-tau217, pg/ml	0.49 [0.29;0.83]	1.13 [0.71;1.40]	0.50 [0.35;0.77]	0.28 [0.24;0.38]	< 0.001
GFAP, pg/ml	117.00 [75.80;142.00]	193.00 [143.50;249.00]	136.00 [116.00;188.50]	126.30 [84.10;166.00]	< 0.001
Nfl, pg/ml	22.80 [19.10;28.50]	29.70 [23.00;43.75]	36.40 [25.60;52.25]	71.45 [61.00;171.00]	< 0.001

*CU C*ognitively unimpaired, *ADCI* Alzheimer’s disease-type cognitive impairment, *SVCI* Subcortical vascular cognitive impairment, *FTD* Frontotemporal dementia, *APOE* Apolipoprotein E, *MMSE* Mini-mental state examination, *Aβ* β-amyloid, *PET* Positron emission tomography, *P-tau217* Phosphorylated-tau217, *GFAP* Glial fibrillary acidic protein, *NfL* Neurofilament light chain.

### Distribution of AT biological stages by clinical stages across diagnosis

**Fig. 1:**
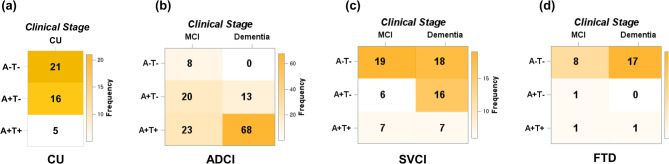
Frequency heatmaps of biological and clinical stages in (**a**) CU, (**b**) ADCI, (**c**) SVCI, and (**d**) FTD.

In ADCI (Fig. 1b), the MCI stage included 39.2% (20/51) A + T − and 45.1% (23/51) A + T+, while the dementia stage consisted of 16.0% (13/81) A + T − and 84.0% (68/81) A + T+. In SVCI (Fig. 1c), the AT biological stages were less advanced, with the MCI stage showing 18.8% (6/32) A + T − and 21.9% (7/32) A + T+, while the dementia stage had 39.0% (16/41) A + T − and 17.1% (7/41) A + T+. In FTD (Fig. 1d), the effect was more pronounced, with 80.0% (8/10) A − T− in MCI and 94.4% (17/18) A − T− in the dementia stage.

### Comparison in Aβ and tau PET uptakes across diagnosis within the same AT stage

**Fig. 2:**
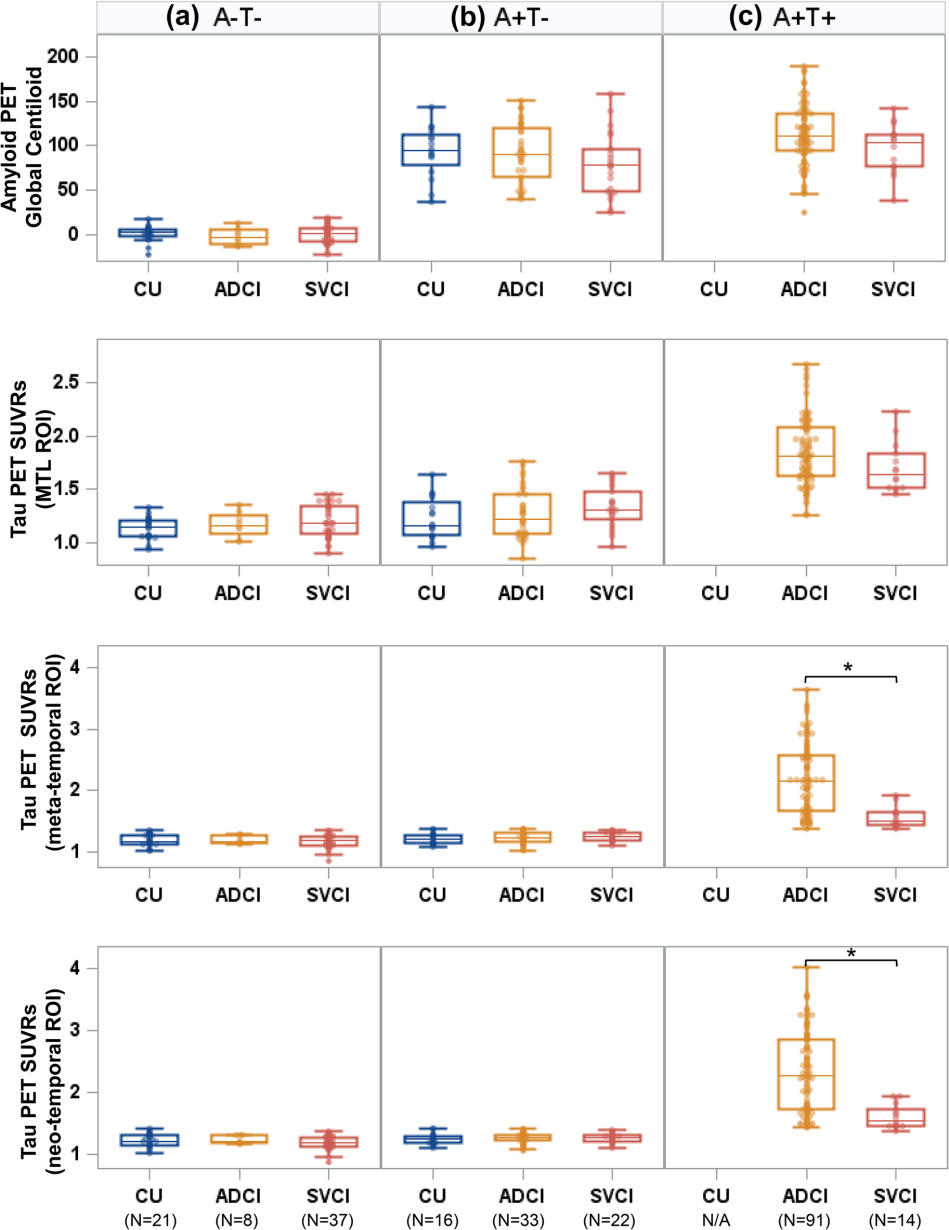
Comparison in AT PET uptakes across diagnosis within the same AT stage: (**a**) A − T−, (**b**) A + T−, and (**c**) A + T+. The groups with five or fewer participants were excluded. Thus, the FTD and A + T + CU groups were excluded from the comparison.

For each AT biological stage, Aβ and tau PET uptakes were compared across diagnostic groups (Fig. 2). Global Aβ (rdcCL) and medial temporal tau uptakes (SUVR) did not significantly differ across diagnoses within the same AT categories. However, meta-temporal tau (2.156 vs. 1.788, *P* = 0.009) and neocortical tau uptakes (2.281 vs. 1.848, *P* = 0.008) on PET were significantly higher in A + T + ADCI than in A + T + SVCI, indicating more advanced tau pathology in A + T + ADCI than A + T + SVCI despite being in the same A + T + biological stage. Only a small number of FTD participants were classified as A + T − or A + T+, and thus were excluded from the analyses.

### Plasma biomarker profiles according to biological stages within each diagnostic group

**Fig. 3:**
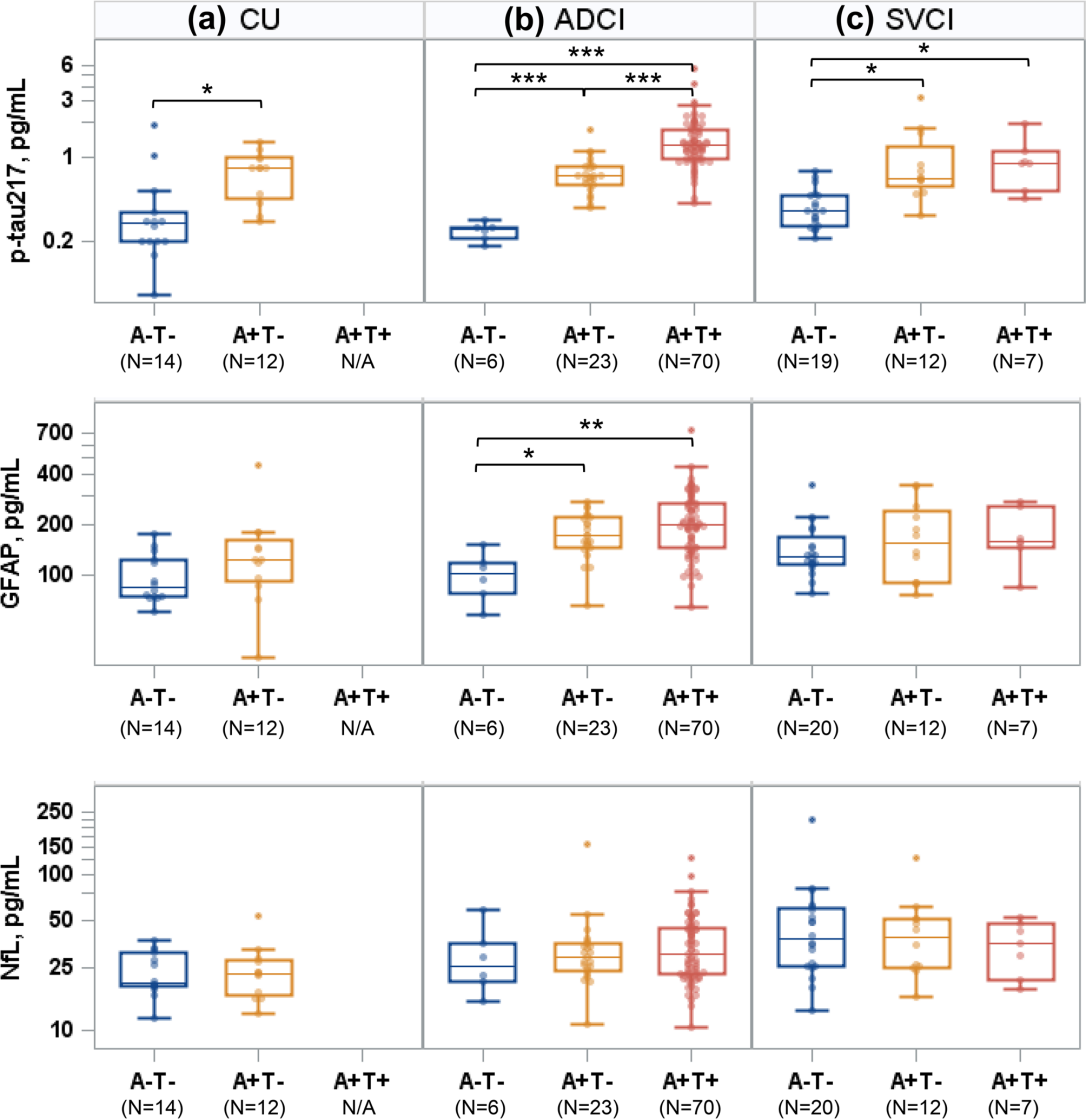
Plasma biomarker profiles according to biological stage within each clinical diagnosis: (**a**) CU, (**b**) ADCI, and (**c**) SVCI. The groups with five or fewer participants were excluded. Thus, the FTD and A + T + CU groups were excluded from the comparison.

P-tau217 levels progressively increased across advancing AT stages within the CU and ADCI groups. However, in the SVCI group, while p-tau217 levels were higher in A + T − compared to A − T− (*P* < 0.001), there was no significant difference between the A + T − and A + T + stages. GFAP levels were significantly higher in both the A + T + and A + T − stages compared to A − T− within the ADCI group, but no significant differences were observed across AT stages in the CU and SVCI groups. NfL levels did not differ significantly across AT stages within each diagnostic group (Fig. 3).

### Plasma biomarker profiles according to diagnosis within each biological stage

**Fig. 4:**
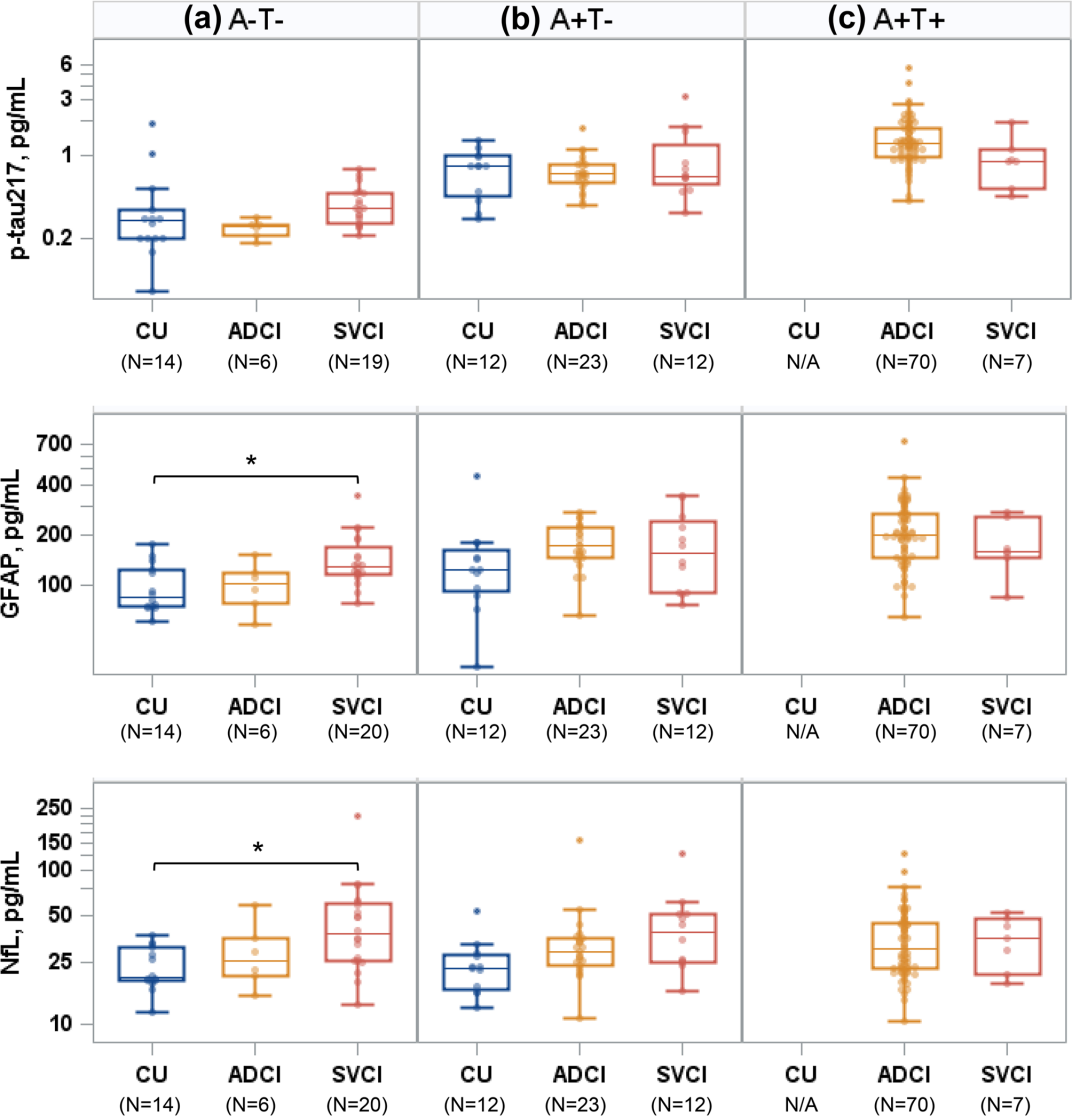
Plasma biomarker profiles according to clinical diagnosis within each biological stage: (**a**) A − T−, (**b**) A + T−, and (**c**) A + T+. The groups with five or fewer participants were excluded. Thus, the FTD and A + T + CU groups were excluded from the comparison. Abbreviations: CU, cognitively unimpaired; ADCI, Alzheimer’s disease-type cognitive impairment; SVCI, subcortical vascular cognitive impairment; FTD, frontotemporal dementia; A, amyloid; T, tau; p-tau217, phosphorylated-tau217; GFAP, glial fibrillary acidic protein; NfL, neurofilament light chain. ^*^, *P* < 0.05; ^**^, *P* < 0.001; ^***^, *P* < 0.0001.

P-tau217 levels showed no significant differences among the CU, ADCI, and SVCI groups in all AT stages, after adjusting for age, *APOE* genotype, and neo-temporal tau PET SUVR (Fig. 4). GFAP levels were significantly higher in the SVCI group compared to the CU group in the A + T − stage (*P* = 0.012). NfL levels were elevated in the SVCI group compared to the CU group in the A − T− stage (*P* = 0.025).

### Cognitive trajectories by diagnosis within the same AT biological stage and diagnostic groups

**Fig. 5:**
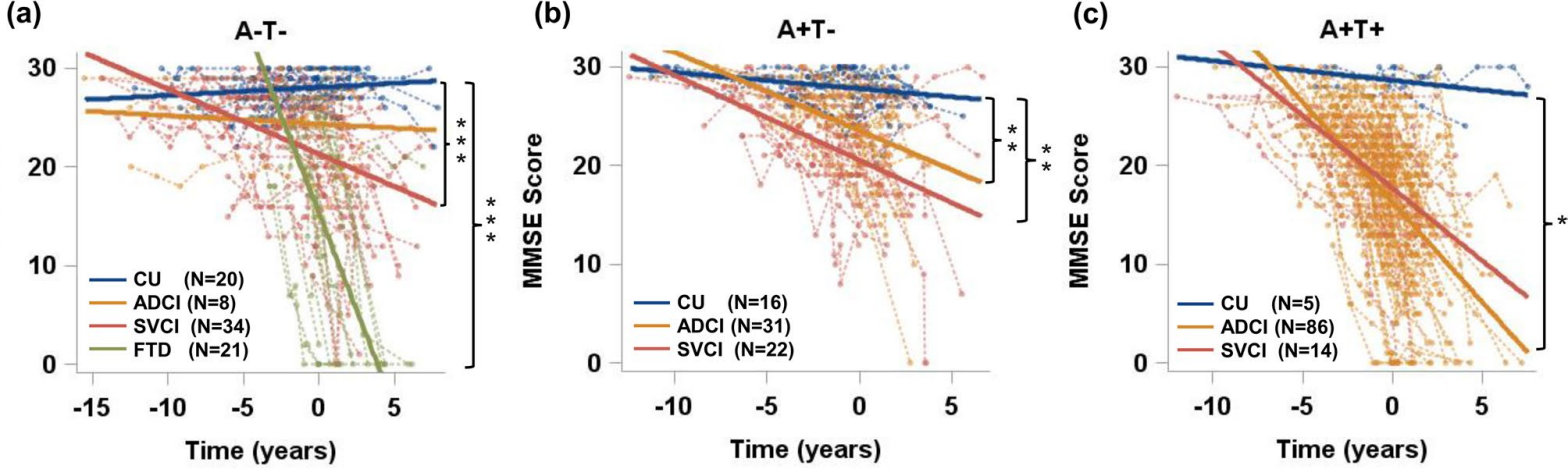
Cognitive trajectories by AT biological stages: (**a**) A − T−, (**b**) A + T-, and (**c**) A + T + stages. The groups with a small sample size (A + T − FTD (*n* = 1) or A + T + FTD (*n* = 2)) were excluded from analysis. Linear mixed-effects models were applied to evaluate interactions between time and staging groups on the repeated measures of MMSE, adjusting for age, education level, *APOE* genotype, and SUVRs in neo-temporal ROI.

In the A − T− stage, both SVCI and FTD participants exhibited more rapid deterioration in MMSE scores compared to CU participants (SVCI vs. CU, β = −0.73, *P* = 0.002; FTD vs. CU, β = −3.97, *P* < 0.001; Fig. 5 and Supplementary Table**)**. No significant differences were observed between ADCI and CU. In the A + T − stage, both ADCI and SVCI participants experienced faster deterioration in MMSE scores compared to CU participants (ADCI vs. CU, β = −0.64, *P* = 0.004; SVCI vs. CU, β = −0.67, *P* = 0.004). In the A + T + stage, only ADCI participants showed a significantly faster decline in MMSE scores compared to CU participants (β = −1.83, *P* = 0.013).

**Fig. 6:**
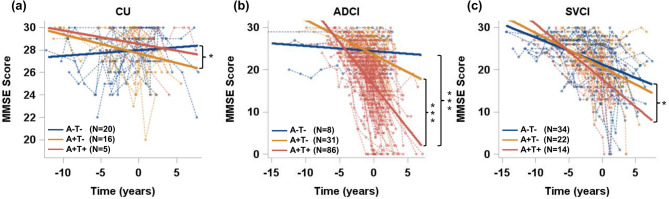
Cognitive trajectories by AT stages within each diagnostic group: (**a**) CU, (**b**) ADCI, and (**c**) SVCI groups. The groups with a small sample size (A + T − FTD (*n* = 1) or A + T + FTD (*n* = 2)) were excluded from analysis. Linear mixed-effects models were applied to evaluate interactions between time and staging groups on the repeated measures of MMSE, adjusting for age, education level, and *APOE* genotype.

Cognitive trajectories by AT stages within each diagnostic group were additionally assessed; results are presented in Fig. 6. A + T − CU participants showed faster decline than A − T− CU. In ADCI, A + T + showed significantly more rapid deterioration than both A − T− and A + T−. In SVCI, A + T + showed faster decline than A − T− and demonstrated a trend toward a faster decline compared to A + T−.

## Discussion

This study examined the distribution of AT biological stages by clinical stage and explored plasma biomarkers and cognitive trajectories according to AT biological stage across various cognitive impairment groups. Our main findings were as follows: (1) Compared to ADCI participants, SVCI and FTD participants exhibit more advanced clinical stages at equivalent AT stages, with FTD participants notably showing an advanced clinical stage even in the A − T− stage. (2) In the A − T− stage, nonspecific AD biomarkers, such as plasma GFAP and NfL levels, were elevated in SVCI participants compared to CU participants. (3) In the A − T− stage, cognitive decline was more pronounced in SVCI and FTD participants compared to CU participants. However, in the A + T − and A + T + stages, cognitive trajectories in ADCI and SVCI participants became indistinguishable, possibly due to the strong influence of AD pathology overriding vascular contributions. Taken together, our findings suggest that non-AD pathologies contribute to more advanced clinical stages at equivalent AT biological stages compared to ADCI and are associated with elevated nonspecific plasma biomarkers and greater cognitive decline, particularly in the A − T− stage. These findings underscore the importance of considering non-AD processes when interpreting AD biomarkers in clinical and research settings.

Our first major finding was that SVCI and FTD participants exhibited more advanced clinical stages compared to ADCI participants, relative to biological stages, with SVCI participants showing a notable mismatch and an even more pronounced discrepancy in FTD participants. Specifically, the MCI and dementia stages were prevalent in SVCI patients despite minimal or absent AD biomarker elevation (A − T− and A + T−), suggesting a weak association between cognitive impairment and AD pathology. This trend was even stronger in FTD participants, where severe cognitive decline occurred at the lowest AT stage (A − T−). These findings align with previous studies demonstrating that non-AD dementias, such as FTD and SVCI, can progress clinically without corresponding increases in AD biomarkers^3^. In FTD, cognitive impairment appears largely independent of Aβ or tau pathology, while in SVCI, cognitive decline is attributed to vascular factors such as WMH, which are only weakly correlated with AD biomarkers. These patterns emphasise the need for biomarkers beyond AD pathology to accurately assess disease severity in dementia subtypes. Reflecting this, the 2024 Revised Criteria^2^ advocate for an integrated biological and clinical staging approach that accounts for co-pathologies. While AD typically follows a predictable biomarker trajectory, our findings for SVCI and FTD highlight the importance of considering both AD and non-AD pathologies for an accurate assessment of disease progression.

Our second major finding was that, in the A − T− stage, nonspecific AD markers, i.e., plasma GFAP and NfL, were significantly elevated in SVCI compared to CU, and remained relatively stable across all AT stages within the SVCI group. These findings suggest that SVCI is associated with elevated markers of neuroinflammation and neurodegeneration independent of AD pathology. In line with previous studies^34–36^, our findings further support that plasma p-tau217 serves an AD-specific biomarker, with levels increasing in association with Aβ across all diagnostic groups and with tau particularly in the ADCI group. In contrast, GFAP and NfL exhibited a more nonspecific pattern. Pathological analyses of cerebral small vessel disease have shown dense GFAP-immunoreactive astrocytes in periventricular white matter^37^ and in clusters within the basal ganglia and thalamus, suggesting that plasma GFAP may reflect blood-brain barrier disruptions and proximity to cerebrospinal fluid and the glymphatic system^38^, linking GFAP to cerebrovascular pathology in SVCI. Similarly, earlier studies have reported elevated levels of GFAP and NfL in cerebrovascular conditions^39–43^, with GFAP indicating astrocytic activation^44^ and NfL signalling neuronal injury^45^. However, in the A + T − or A + T + stages, the elevations of GFAP and NfL in SVCI compared to other diagnostic groups were less prominent. This may be because both vascular and AD pathologies contribute to the increases in these nonspecific biomarkers. Notably, previous studies have shown that GFAP increases in response to Aβ-dependent neuroinflammation^34,46^, consistent with our finding that GFAP levels were higher in A + T + and A + T − ADCI compared to A − T− ADCI. Collectively, these findings suggest that multiple pathological processes contribute to elevations in nonspecific biomarkers, highlighting the need for further studies to disentangle the independent effects of vascular and AD pathologies on neuroinflammation and neurodegeneration.

Our final major finding was that in the A − T− stage, cognitive decline was more pronounced in SVCI and FTD participants compared to CU participants, reflecting the significant impact of non-AD pathologies on cognitive impairment. However, in the A + T − and A + T + stages, cognitive trajectories in ADCI and SVCI participants became indistinguishable. Several explanations may account for this finding. First, AD pathology may exert a dominant effect, particularly in the A + T + stage, thereby overshadowing the influence of coexisting non-AD pathologies. Increasing Aβ and tau accumulation may intensify neurodegeneration and cognitive decline to the extent that additional contributions from vascular pathology become less detectable. Second, selection bias may contribute to this finding. We observed that the A + T + SVCI group exhibited lower tau uptake compared to A + T + ADCI group, despite both groups meeting the criteria for the same AT stage. Given that A + T + SVCI participants have both vascular and AD pathologies contributing to cognitive impairment, even a lower burden of AD pathology may prompt clinical presentation. Alternatively, participants with mixed pathologies may have experienced rapid clinical deterioration, leading to early institutionalization or dropout and consequently being underrepresented in our cohort. Therefore, further studies with larger sample sizes are needed to clarify the relative contributions of AD and non-AD pathologies to cognitive decline.

The strength of our study lies in its integrative analysis of multiple biomarkers and cognitive trajectories across various types of dementia. However, the study has some limitations. First, the lack of pathological confirmation limits the interpretation of the observed biomarker patterns in non-AD dementias and raises the possibility of false-positive or false-negative Aβ PET findings, particularly in the presence of cerebral atrophy. Second, the relatively small number of FTD participants who underwent tau PET imaging, particularly in the A + T − and A + T + stages, limited the analysis of plasma biomarkers and clinical trajectories; therefore, these strata are reported descriptively with exact n indicated and interpreted as hypothesis-generating, underscoring the need for confirmation in larger, multicenter cohorts. Third, our study demonstrated a relatively low proportion of dementia cases in the A + T + SVCI group. This may be attributed to faster disease progression, leading to earlier admission to nursing facilities and loss to follow-up, resulting in fewer A + T + SVCI participants being captured at the dementia stage. Therefore, future study designs should address this potential selection bias, as attrition of patients in the earlier stages of A + T + SVCI may limit the accurate characterization of the disease trajectory. Fourth, the MMSE was used as a pragmatic global measure, but it does not fully capture disease-specific cognitive dynamics; therefore, cognitive trajectories should be interpreted with caution. Our primary inferences were based on comparisons within fixed A/T stages to reduce potential confounding by underlying pathology. Fifth, within ADCI, there were no dementia participants at the A − T− stage. However, this pattern was expected and reflects clinical logic rather than selection bias. All clinically diagnosed ADCI participants underwent amyloid and tau PET; those classified as A − T− were MCI only, and ADCI dementia is highly unlikely to be biologically A − T−. Finally, our cross-sectional design limited our ability to fully capture longitudinal changes in biomarker levels and cognitive decline, which would provide a more comprehensive understanding of disease progression over time. Nevertheless, this study is novel in demonstrating integrated biological and clinical staging, downstream plasma biomarkers, and cognitive trajectories in relation to AD biological staging across various dementias, offering valuable insights into dementia subtype differentiation and progression.

## Conclusions

This study provides a comprehensive analysis of the distribution of AT biological and clinical stages across ADCI, SVCI, and FTD. Additionally, we identify differences in plasma biomarkers and cognitive trajectories according to biological stages in these dementias. These findings offer valuable insights into the disease mechanisms and progression of non-AD dementias, enhancing our understanding of how AD biological staging interacts with other pathological processes in these conditions.

## Supplementary Information

Below is the link to the electronic supplementary material.

Supplementary Material 1

## Data Availability

The anonymised data for the analyses presented in this report are available upon request from the corresponding authors.

## Abbreviations

CU: Cognitively unimpaired
ADCI: Alzheimer’s disease-type cognitive impairment
SVCI: Subcortical vascular cognitive impairment
FTD: Frontotemporal dementia
A: Amyloid
T: Tau
MCI: Mild cognitive impairment
SUVR: Standardized uptake value ratio
SVCI: Subcortical vascular cognitive impairment
FTD: Frontotemporal dementia
ROI: Region of interest
p-tau217: Phosphorylated-tau217
GFAP: Glial fibrillary acidic protein
NfL: Neurofilament light chain
MMSE: Mini-mental state examination
PET: Positron emission tomography
