# Supplementary material for: Integrative analysis of AT classification, plasma biomarkers, and cognitive trajectories across diverse dementia syndromes
Source: Sci Rep. 2025 Dec 11;16:610. doi: 10.1038/s41598-025-30117-y (PMC12775461; doi:10.1038/s41598-025-30117-y)
Supplement: Supplementary file 1 — Supplementary Material 1 [file 41598_2025_30117_MOESM1_ESM.docx]

**SUPPLEMENTARY MATERIALS**

**Manuscript ID**: 13f4db6a-d501-40b0-a4ba-840bcbed9d98

**Title**: Integrative analysis of AT classification, plasma biomarkers, and cognitive trajectories across diverse dementia syndromes

**Authors**: Jihwan Yun, MD, PhD^1#^, Min Young Chun, MD, PhD^2,3#^, Henrik Zetterberg, MD, PhD ^4,5,6,7,8,9^, Kaj Blennow, MD^4,5,10,11^, Fernando Gonzalez-Ortiz, MD^4,5^, Nicholas J. Ashton, PhD^4,12,13,14^, Daeun Shin, MD, MSc^15^, Soyeon Yoon, MD^15^, Heejin Yoo, MSc^15^, Jun Pyo Kim, MD, PhD^15^, Hongki Ham, MSc^15^, Yuna Gu, MSc^15^, Hee Jin Kim, MD, PhD^15,16,17^, Seung Hwan Moon, MD, PhD^18^, Hanna Cho, MD, PhD^19^, Jae Yong Choi, PhD^20^, Byung Hyun Byun, PhD^21^, Su Yeon Park, MD^22^, Jeong Ho Ha, MD, PhD^22^, Duk L. Na, MD, PhD^23^, Sang Won Seo, MD, PhD^15,16,17*^, Hyemin Jang, MD, PhD^24*^ & on behalf of the K-ROAD study groups

**Supplementary Table.** Cognitive Trajectories by Biological Stages

| 1. **A–T–** | | | | | |
| --- | --- | --- | --- | --- | --- |
| **Model** | **Group Comparison** | **beta** | **SE** | ***P*-value** | **adjusted *p*-value^*^** |
| **Model 1** |  |  |  |  |  |
| (with ε4) | CU vs. ADCI | -0.140 | 0.337 | 0.678 | 0.678 |
|  | CU vs. SVCI | -0.728 | 0.223 | 0.001 | 0.002 |
|  | CU vs. FTD | -3.972 | 0.287 | 0.000 | 0.000 |
|  | ADCI vs. SVCI | -0.588 | 0.315 | 0.063 | 0.076 |
|  | ADCI vs. FTD | -3.832 | 0.363 | 0.000 | 0.000 |
|  | SVCI vs. FTD | -3.244 | 0.261 | 0.000 | 0.000 |
| **Model 2** |  |  |  |  |  |
| (without ε4) | CU vs. ADCI | -0.139 | 0.336 | 0.679 | 0.679 |
|  | CU vs. SVCI | -0.729 | 0.223 | 0.001 | 0.002 |
|  | CU vs. FTD | -3.971 | 0.287 | 0.000 | 0.000 |
|  | ADCI vs. SVCI | -0.590 | 0.315 | 0.062 | 0.074 |
|  | ADCI vs. FTD | -3.832 | 0.363 | 0.000 | 0.000 |
|  | SVCI vs. FTD | -3.243 | 0.261 | 0.000 | 0.000 |

| 1. **A+T–** | | | | | | 1. **A+T+** | | | | | |
| --- | --- | --- | --- | --- | --- | --- | --- | --- | --- | --- | --- |
| **Model** | **Group Comparison** | **beta** | **SE** | ***P*-value** | **adjusted**  ***p*-value^*^** | **Model** | **Group Comparison** | **beta** | **SE** | ***p*-value** | **adjusted**  ***p*-value** |
| **Model 1** |  |  |  |  |  | **Model 1** |  |  |  |  |  |
| (with ε4) | CU vs. ADCI | -0.640 | 0.208 | 0.002 | 0.003 |  | CU vs. ADCI | -1.834 | 0.638 | 0.004 | 0.013 |
|  | CU vs. SVCI | -0.674 | 0.215 | 0.002 | 0.003 |  | CU vs. SVCI | -1.365 | 0.713 | 0.056 | 0.084 |
|  | ADCI vs. SVCI | -0.033 | 0.189 | 0.860 | 0.860 |  | ADCI vs. SVCI | 0.469 | 0.374 | 0.211 | 0.211 |
| **Model 2** |  |  |  |  |  | **Model 2** |  |  |  |  |  |
| (without ε4) | CU vs. ADCI | -0.622 | 0.207 | 0.003 | 0.004 |  | CU vs. ADCI | -1.809 | 0.639 | 0.005 | 0.014 |
|  | CU vs. SVCI | -0.669 | 0.213 | 0.002 | 0.004 |  | CU vs. SVCI | -1.312 | 0.713 | 0.066 | 0.100 |
|  | ADCI vs. SVCI | -0.047 | 0.187 | 0.802 | 0.802 |  | ADCI vs. SVCI | 0.498 | 0.375 | 0.185 | 0.185 |

Cognitive trajectories by AT biological stages: Longitudinal changes in MMSE scores across binary AT PET staging groups based on PET imaging. Linear mixed-effects models were applied to evaluate interactions between time and staging groups on the repeated measures outcome MMSE, adjusting for age, education level and SUVRs in the neo-temporal ROI with ε4 (model 1) or without ε4 (model 2): (a) A–T–, (b) A+T–, and (c) A+T+ groups.

Groups with small sample sizes (A+T– FTD (*n* = 1) and A+T+ FTD (*n* = 2)) were excluded from the analysis.

Abbreviations: CU, cognitively unimpaired; ADCI, Alzheimer’s disease-type cognitive impairment; SVCI, subcortical vascular cognitive impairment; FTD, frontotemporal dementia; A, amyloid; T, tau; MMSE, Mini-Mental State Examination; PET, positron emission tomography; SUVR, standardised uptake value ratio; ROI, region of interest.
